# T Cells Targeting SARS-CoV-2: By Infection, Vaccination, and Against Future Variants

**DOI:** 10.3389/fmed.2021.793102

**Published:** 2021-12-24

**Authors:** Thi H. O. Nguyen, Carolyn A. Cohen, Louise C. Rowntree, Maireid B. Bull, Asmaa Hachim, Katherine Kedzierska, Sophie A. Valkenburg

**Affiliations:** ^1^Department of Microbiology and Immunology, The Peter Doherty Institute for Infection and Immunity, The University of Melbourne, Melbourne, VIC, Australia; ^2^HKU-Pasteur Research Pole, Li Ka Shing Faculty of Medicine, School of Public Health, The University of Hong Kong, Pokfulam, Hong Kong SAR, China

**Keywords:** SARS-CoV-2, T cells, T follicular cell, tetramer, immunodominance, crossreactivity

## Abstract

T cell responses are a key cornerstone to viral immunity to drive high-quality antibody responses, establishing memory for recall and for viral clearance. Inefficient recruitment of T cell responses plays a role in the development of severe COVID-19 and is also represented by reduced cellular responses in men, children, and diversity compared with other epitope-specific subsets and available T cell receptor diversity. SARS-CoV-2-specific T cell responses are elicited by multiple vaccine formats and augmented by prior infection for hybrid immunity. Epitope conservation is relatively well-maintained leading to T cell crossreactivity for variants of concern that have diminished serological responses.

## Introduction

The SARS-CoV-2 virus has rapidly spread globally to cause the COVID-19 pandemic due to a lack of pre-existing neutralizing antibodies and viral shedding during presymptomatic infection. Whilst the virus has caused global disruption, millions of deaths, and long-term morbidity, the majority of infections are asymptomatic. A coordinated SARS-CoV-2-specific cellular response of B and T cells and the development of neutralizing antibodies are coincident to recovery from infection (1, 2) and limit immunopathology (3) to form long-term memory (4) to mitigate reinfection. The SARS-CoV-2 virus has a unique inflammatory signature compared with other virus infections (5, 6) and encodes a number of proteins with immunomodulatory function to regulate cellular trafficking, cytokine responses, and major histocompatibility complex (MHC) class I [reviewed in (7)]. Furthermore, a wide range of COVID-19 vaccine efficacy from infection is reported and coincident with neutralizing antibody titres (8), whilst severe infection and morbidity are substantially reduced by all vaccine platforms (9) despite lower neutralizing titres (10) suggesting that immune responses beyond neutralizing titres can protect from severe COVID-19, such as T cell immunity. This review will focus on the unique nature of the T cell response elicited by the pandemic SARS-CoV-2 virus during a primary immune response by infection and vaccination, and the relationship to common cold coronaviruses (CCCoVs) and variants of concern (VoC).

### The Origins of SARS-CoV-2 T Cell Peptides During Infection

**Figure 1:**
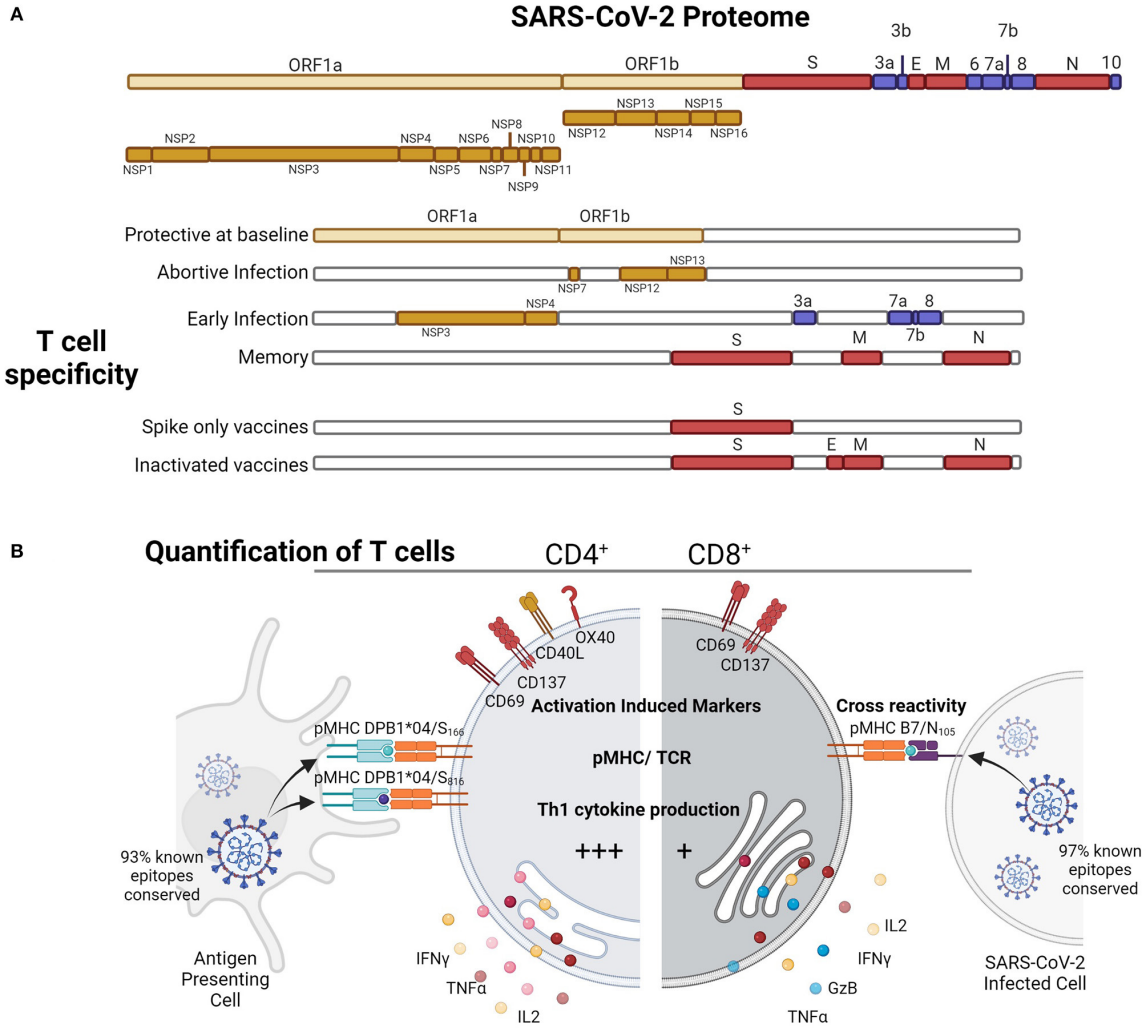
SARS-CoV-2-specific T cell responses to various proteins at different stages of exposure **(A)** in infection and vaccination can be quantified by **(B)** activation induced markers, pMHC tetramer or multimer binding for known immunodominant epitopes, or by cytokine induction **(B)**. Figure produced with Biorender.

SARS-CoV-2 belongs to the β-coronaviruses genus, which is closely related to SARS-CoV and MERS-CoV viruses which have had limited circulation in humans causing severe infections during outbreaks [reviewed in (11)]. Other human β-coronaviruses, such as HCoV-HKU-1 and HCoV-OC43, are CCCoVs which only causes minor disease (12, 13), but most adults have been exposed and repeated infection occurs. SARS-CoV-2 has a 27 kb positive-sense, single-stranded RNA and non-segmented genome, with half the genome encoded by the open reading frame (ORF) 1a/b which is cleaved to at least 16 non-structural proteins (nsp) which function for replication and host evasion [reviewed in (14)] (Figure 1A). The virion is encoded by structural proteins: Spike (S), Envelope (E), Matrix (M), and Nucleocapsid (N), whilst accessory protein ORFs 3a, 3c, 3d-2, 6, 7a, 7b, 8, N, and 9b support virus replication; virion formation, and some proteins such as M and ORF3a can be expressed at the membrane surface due to interactions with the autophagolysosome pathway (15). Due to the linear nature of the SARS-CoV-2 ssRNA genome, upon infection, there is immediate 5′ translation of replication transcription complex (RTC) of nsp7, nsp12, and nsp13 to ensure virus replication. 3′ transcription leads to the highest abundance of N transcripts and ultimately protein, and due to genome location and ribosome density, the next most abundant transcripts early after infection are M > ORF7a > ORF3a > ORF8 > ORF6 (16), which is close to HLA-I peptide presentation during *in vitro* infection of HEK293-T and A549 cell lines, with the most abundant surface peptides derived from N > M > ORF9b > ORF3a = S proteins (17). Hence, the linear and positive-sense genome of SARS-CoV-2 shapes the T cell response.

### Crossreactive T Cell Responses by SARS-CoV-2 Infection: Getting Ahead From the Baseline Race

SARS-CoV-2 T cells have been quantified by a number of different immune assays based on epitope presentation leading to T cell receptor binding or activation for upregulation of surface markers or cytokine secretion (Figure 1B). The characterization of functional antiviral cytokine and activated T cells elicited by SARS-CoV-2 infection by *in vitro* stimulation has used either HLA optimized peptide “megapools” (18, 19), selected expected crossreactive peptides (20), comprehensive peptidome with the omission of ORF1 (21), or whole peptidome functional pools (22). Antigen-specific responses once a peptide epitope has been identified (23) can also be quantified by pMHC binding using tetramers or multimers which is useful for downstream cellular characterization (23–28). Furthermore, antigen-specific responses have also been identified by mapping of HLA presented peptides during *in vitro* infection to reveal cryptic T cell epitopes within proteins that are boosted by recent infection in patients with COVID-19 (17), which can be ORF independent, and therefore cryptic epitopes can be generated during infection (19). Therefore, the definition of SARS-CoV-2 T cells is assay-dependent and contingent upon the epitopes included; however, consistent trends amongst studies with different approaches have shown that robust T cell responses are generated by SARS-CoV-2 infection (Table 1).

**Table 1:** SARS-CoV-2 CD4^+^ and CD8^+^ T cell responses in infection and vaccination.

		**CD4^**+**^ T cell responses**	**Ref**.	**CD8^**+**^ T cell responses**	**Ref**.
Infection	Adults	−100% recovered patients have activated cells—Produce IFNγ, TNFα, IL2 - Express CD134, CD137 - Recruited early during acute infection - HLA-DPB1*04/S_166−180_ is a dominant SARS-CoV-2-specific epitope following infection	(18, 21, 22, 28, 29)	−70% recovered patients activated cells - Produce IFNγ, TNFα, IL2, GzB - Express CD69, CD137 - Recruited later during infection at convalescence - HLA-B*07:02/N_105−113_ is a dominant SARS-CoV-2-specific epitope following infection (38 × larger than B7/N_257_, A2/S_269_ and A24/S_1208_)	(18, 21, 22, 26, 29–32)
	Children	- Reduced response magnitude compared with adults - T naïve in children > T naïve in adults	(22)	- Reduced response magnitude compared with adults	(22)
	Severe	- Reduced structural response - Hyperactivated CD25^+^ CD4^+^ T cells expressing furin - Increased T_FH_2, reduced T_FH_1 and T_FH_17	(33, 34)	- Reduced response - Less exhaustion, defined by coexpression of PD-1, CD38 and HLA-DR than mild patients.	(33, 35)
Vaccination		- Induction of strong CD4^+^ responses across most vaccine platforms - No significant differences found in studies comparing two vaccines in parallel	(36–40)	- Induction of CD8^+^ responses across most vaccine platforms (at lower levels than CD4^+^ T cell fold changes)	(36–40)
Hybrid immunity		- Increased responses compared with infection induced immunity alone. - One dose of mRNA vaccination boosts previous response from natural infection or ChAdOx1 nCov-19 vaccination - Two doses did not boost responses further	(41, 42)	- mRNA vaccination boosts previous response after one dose - Further boosting is seen after two doses alongside increased memory responses	(42)
Crossreactivity Infection		- Unexposed healthy individuals have crossreactive CD4^+^ T cells against ORF1a/b nsp7 and nsp13 proteins - Dominant epitopes DPB1*04/S_166_ and /S_186_ are conserved in currently known VoCs	(20, 28, 43, 44)	- Dominant epitope B7/N_105_ epitope is conserved in currently known VoCs	(21, 26, 32, 45)
	Vaccination	- mRNA vaccination induces CD4^+^ T cells crossreactive to several VoCs - Back boosting of HLA-DPB1*04/S_816−830_ responses by mRNA vaccination. Found in 20% unexposed positive, 50% patients and 97% vaccinees. - 7% of epitopes affected in VoC, crossreactivity maintained	(36, 39, 46, 47)	- mRNA vaccine recipients maintain crossreactive CD8^+^ - 3% of epitopes affected in VoC, crossreactivity maintained	(36, 39)

SARS-CoV-2 T cell epitope immunodominance hierarchies are evident (19, 20, 22, 26, 43), which may be attributable to peptide homology with CCCoV (48, 49) and the relative expression of structural, nsp, and accessory proteins during virus SARS-CoV-2 replication (21). The magnitude of SARS-CoV-2 T cell responses generated during infection can also be dictated by the efficiency of T cell recruitment by crossreactivity and avidity (50) and naïve precursor frequency as evident by T cell receptor diversity (26).

Due to the widespread prevalence of CCCoV exposure, 20–60% unexposed individuals (18, 30) display low magnitude of SARS-CoV-2 crossreactive T cells which may provide a baseline of immunity against SARS-CoV-2 (51). These crossreactive responses are mostly reported in the ORF1a/b non-structural proteins (18), and the magnitude of the response has a significant correlation with reduced duration of infection (20). The majority of pre-existing crossreactive T cell responses map to the ORF1a/b (43, 44), especially nsp7 and nsp13 which have high homology to other CoVs (48) (Table 1). SARS-CoV-2 crossreactive T cell responses have also been detected despite lower epitope homology (<67%) (43), determined by PBMC stimulation with overlapping peptide pools. Further work is needed to understand whether these “pre-existing” T cells in unexposed individuals are of a memory (rather than naïve) phenotype directly *ex vivo* and whether they can be efficiently recruited following SARS-CoV-2 infection to provide some level of protection during acute disease.

Epitope-specific T cells for SARS-CoV-2 are derived from different viral proteins at baseline, subclinical infection, early during infection, and long-term memory (Figure 1A). In potentially abortive infections in highly exposed health care workers who remain S/N seronegative (52), RCT (nsp7/12/13)-specific T cells are elevated suggesting T cell boosting with subclinical infection, possibly due to early RCT expression without virion formation. Whilst early after mild infection ORF7 and ORF8 (53), and nsp3, nsp4, ORF3a (18)-specific CD4^+^ T cells are expanded, ultimately the structural responses, S, N and M, become the most immunodominant following infection to convalescence (20, 21, 43). Furthermore, in naïve mouse models during infection (54), mapping by peptides and virus-like particles of structural (S, N, M, E) and selected ORFs (ORF3a, ORF6, ORF7a, ORF8, ORF9b, and ORF9c) showed a similar immunodominance of CD4^+^ T cell responses toward S, N, and ORF8-derived peptides. Overall, the protein specificity of the SARS-CoV-2 T cell response is shaped by prior immunity and the viral lifecycle to race for MHC presentation based on timing and abundance.

### Approaches for Quantification of SARS-CoV-2-Specific T Cells

Early studies utilized HLA class I and class II predicted peptide “megapools” to stimulate PBMCs from COVID-19 individuals (18). Using T cell receptor (TCR)-dependent activation induced marker (AIM) assay, SARS-CoV-2-reactive CD4^+^ (CD134^+^CD137^+^) and CD8^+^ (CD69^+^CD137^+^) T cells (Figure 1B) directed toward S, M, N and other ORFs were detected in 100% and 70% of convalescent patients with COVID-19, respectively (18, 29) (Table 1). The presence of SARS-CoV-2-reactive CD4^+^ and CD8^+^ T cells was also found in acute patients with COVID-19 admitted to ICU at similar frequencies (30). Both SARS-CoV-2-reactive CD4^+^ and CD8^+^ T cell responses were positively associated with RBD IgG antibodies and less severe disease; however, these correlations were lost in individuals older than 65 years (29). Stimulation of PBMCs from patients with COVID-19 with SARS-CoV-2 overlapping peptides also leads to IFN-γ production and clonal expansion of SARS-CoV-2-specific CD8^+^ and CD4^+^ T cells *in vitro* (Figure 1). The CD4^+^ T cell response has been typically more robust than CD8^+^ T cell responses (24, 55, 56).

The magnitude and specificity of the SARS-CoV-2 T cell response is dependent on time, age (22), gender (57), and severity (29) (Table 1). Due to a difference in inflammatory milieu, SARS-CoV-2 T cell responses are increased in women compared with men, and furthermore within men, there is a negative association of T cell response magnitude with age and severity (57). The magnitude of the SARS-CoV-2 memory T cell response in recovered children is significantly reduced compared with adults, with a greater proportion of naïve CD4^+^ T cells indicating incomplete expansion and recruitment of structural-specific T cells (22), which may leave children susceptible to reinfection, albeit hopefully with mild or asymptomatic infection. In mild cases of infection, the CD4^+^ T cell response is recruited early during acute infection (>14 days), whilst the CD8^+^ T cell response lags behind and is recruited later (22). There is correlation across a range of adaptive immune parameters, that is, a seropositive individual is likely to also show cellular immunity; however, with increased age over 65, there is a lack of a coordinated adaptive response, which becomes even more prominent in those over 75, leading to correlation of age and peak disease severity for T cell responses (29). Therefore, various physiological traits impact SARS-CoV-2 cellular immunity.

### Physiological Factors That Influence SARS-CoV-2 T Cell Responses

Immunological misfiring of T cell populations during severe COVID-19 infection has been implicated in the exacerbation of disease (33). Profiling of CD4^+^ T cells after *in silico* sorting by RNA-Seq showed a highly activated CD4^+^ population expressing immunomodulatory marker FoxP3 of T regulatory cells, alongside unconventional differentiation pathways (34). Kalfaoglu et al. have proposed that CD4^+^ T cells have impaired FoxP3-mediated negative feedback leading to a hyperactivated CD25^+^ CD4^+^ T cell population which expressed higher levels of Th2-related genes IL4R and macrophage-associated factors (34). These findings support another study which characterized T cell phenotypes in patients with COVID-19 and found that Th1 and Th17 subsets were reduced in severe disease (34). The ratio of Th1 T follicular helper (T_FH_1) subsets was lower than that of Th2 T_FH_ populations, suggesting that improper T_FH_ differentiation in severe patients may promote Th2 skewed antiinflammatory response that is incapable of effectively managing viral infection (34). Hyperactivated CD4^+^ CD25^+^ T cells express furin which leads to elevated by TCR signaling in severe COVID-19 infection (34) and may assist SARS-CoV-2 viral entry into host cells for a negative feedback loop exacerbating infection (58) (Table 1). Therefore, several viral factors and the immune milieu impact cellular immunity during SARS-CoV-2 infection.

### Tracking Recruitment Efficiency of SARS-CoV-2 Epitope-Specific CD8+ T Cell Responses by TCR Signatures

Impaired dendritic cell function in SARS-CoV-2 infection has been postulated to underlie perturbed proliferation of CD8^+^ T cells (55). The lag in recruitment for SARS-CoV-2 killer CD8^+^ T cells during primary infection of adults may play a role in the long viral shedding and retention of viral antigen (31), particularly for accessory proteins for which no CD8^+^ T cell responses are above baseline (18). Immune misfiring within the CD8^+^ T cell compartment is linked with severity, with severe pathogenesis corresponding to an altered exhaustion profile defined by PD-1, CD38, and HLA-DR coexpression (35). Furthermore, the ORF8 protein of SARS-CoV-2 can directly interfere with MHC-I presentation which can reduce dendritic cell priming and CD8^+^ T cell-mediated cytotoxicity of infected cells (59). However, this effect could to be variant dependent (60) as the folding of the ORF8 protein is pivotal to the downstream downregulation of MHC-I, and ORF8 has been a hotspot of viral adaptation [reviewed in (7)].

To determine cellular recruitment efficiency and memory profiles, SARS-CoV-2-specific CD8^+^ T cell epitopes (peptides + MHC) have been identified using both peptide stimulations and peptide-MHC class I tetramer binding (23–28, 61). Identification of SARS-CoV-2 CD8^+^ T cell specificities restricted by prevalent human HLAs, including HLA-A^*^01:01, HLA-A^*^02:01, HLA-A^*^03:01, HLA-A^*^11:01, HLA-A^*^24:02, HLA-B^*^07:02, HLA-B^*^27:05, HLA-B35:01, HLA-B^*^40:01, and HLA-B^*^44:03 allowed us to understand the magnitude and phenotype of SARS-CoV-2-specific CD8^+^ T cells directly *ex vivo* or after *in vitro* stimulation. The frequencies appear to be generally in the range of ~1 to 5 × 10^−5^ in the CD8^+^ T cell set, with HLA-A^*^02:01-restricted SARS-CoV-2 epitopes being of the lowest frequency (24, 26). This can be exemplified by CD8^+^ T cells directed toward the A2/S_269−277_ epitope detected at comparable frequency (~1.3 × 10^−5^) in acute and convalescent HLA-A^*^02:01^+^ patients with COVID-19 (24). Whilst the numbers were 5× higher than naïve A2/${\text{S}}_{269-277}^{+}$CD8^+^ T cells detected in uninfected HLA-A^*^02:01^+^ donors, they were 10-fold lower when compared to frequencies of influenza-specific A2/${\text{M1}}_{58}^{+}$CD8^+^ and Epstein-Barr virus-specific A2/${\text{BMLF}}_{1280}^{+}$CD8^+^ T cells. Direct *ex vivo* phenotypic analysis of A2/${\text{S}}_{269}^{+}$CD8^+^ T cells from COVID-19 convalescent individuals revealed that A2/${\text{S}}_{269}^{+}$CD8^+^ T cells were suboptimally stimulated and contained naïve, stem cell memory and central memory A2/${\text{S}}_{269}^{+}$CD8^+^ T cells rather than effector memory populations; therefore, epitope-specific CD8^+^ T cells are not fully recruited and activated during SARS-CoV-2 infection.

The low precursor frequency of A2/${\text{S}}_{269}^{+}$CD8^+^ T cells can be explained, at least partially, by the skewed TCR repertoire, with common TRBV gene segments (TRBV2, TRBV7-9, and TRBV20-1), TRBJ (TRBJ2-2, TRBJ2-7), TRAV (TRAV12-1, TRAV12-2, TRAV14/DV4), and TRAJ (TRAJ43, TRAJ30) across different patients with COVID-19. Strikingly, two key TCRα motifs in the CDR3α region were detected across COVID-19 individuals, namely TRAV12-1/TRAJ43 CVVN*XXX*DMRF motif paired with different prominent TRBV genes, and TRAV12-2/TRAJ30 CAVN*X*DDKIIF paired with TRBV7-9, which is also found by Shomuradova et al. (62). A subsequent study by Chaurasia et al. determined a ternary structure of the A2/S_269_ complexed with the dominant TRAV12^+^ TCR (63) found from our previous study (26) to show the importance of TRAV12^+^ TCR docking atop HLA-A^*^02:01, with A2/S_269_ recognition being mediated by both TRAV12 germline-encoded residues and amino acids derived from conserved motifs within CDR3α and CDRβ regions. This ternary structure of the SARS-CoV-2-specific TCR provides a molecular basis underlying biased T cell receptor recognition of HLA-A^*^02:01-restricted epitope comprising the peptide derived from the SARS-CoV-2 spike protein.

CD8^+^ T cell responses directed at the prominent HLA-A^*^24:02-restricted S_1208−1216_ epitope appear to be of similar frequency to other multimer-specific CD8^+^ T cells in both patients infected with SARS-CoV-2 and pre-pandemic PBMCs and were characterized by diverse TCRαβ repertoire characterized by a common TCRβ motif across patients with COVID-19 (27). In contrast, the HLA-B^*^07:02-restricted N_105−113_ epitope (B7/N_105_) appears to be the most dominant SARS-CoV-2 CD8^+^ T cell specificity known to date (23, 25, 26, 32) (Figure 1B and Table 1). Our previous direct *ex vivo* analyses in PBMCs from patients with COVID-19, and also pre-pandemic PBMCs, tonsils, lungs, and spleens, assessing CD8^+^ T cells directed at four SARS-CoV-2 epitopes (B7/N_105_, B7/N_257_, A2/S_269_, and A24/S_1208_), demonstrate that B7/${\text{N}}_{105}^{+}$CD8^+^ T cells were immunodominant by up to 38-fold in patients with COVID-19 and pre-pandemic samples, comparing with three subdominant SARS-CoV-2-specific CD8^+^ T cell populations. Given such a high precursor frequency of B7/${\text{N}}_{105}^{+}$CD8^+^ T cells, a question arose whether immunodominant B7/${\text{N}}_{105}^{+}$CD8^+^ T cell responses originated from pre-existing memory B7/${\text{N}}_{105}^{+}$CD8^+^ pools crossreacting with seasonal human coronavirus, or in contrast, represent high frequency naïve B7/${\text{N}}_{105}^{+}$CD8^+^ pools. Interestingly, epitope-specific CD8^+^ T cells directed at all four SARS-CoV-2 CD8^+^ T cell specificities tested were mainly of a naïve phenotype in pre-pandemic donors. Extreme diverse TCRαβ repertoire together with extraordinary plasticity in TCRα-TCRβ pairing and lack of common TRBV, TRBJ, TRAV, or TRAJ gene segments in B7/${\text{N}}_{105}^{+}$CD8^+^ T cell populations underpins such high precursor frequencies and immunodominance of B7/${\text{N}}_{105}^{+}$CD8^+^ T cells. Of an important note is that all of the above SARS-CoV-2-derived peptides comprising CD8^+^ T cell epitopes are predominantly conserved across different VoCs.

Whilst several SARS-CoV-2-specific CD8^+^ T cell epitopes across a number of HLA class I glycoproteins have been described, to date, our knowledge on CD4^+^ T cell epitopes is limited, with HLA-DPB1^*^04/S_166−180_ being the most dominant SARS-CoV-2-specific CD4^+^ T cell epitope described so far in infected individuals (28). Furthermore, sequence homology of T cell epitopes from SARS-CoV-2 with related CCCoV has resulted in back-boosting of crossreactive CD4^+^ T cell responses during SARS-CoV-2 infection and mRNA BNT162b2 vaccination for the S_816−830_ peptide (46). Therefore, the TCR signature and naïve phenotype of epitope-specific SARS-CoV-2 T cell responses imply incomplete recruitment during infection, and more research is needed to determine the mechanisms to improve T cell responses against COVID-19.

### Protective Role of T Cell in COVID-19

Passive transfer studies of immune serum and T cell subsets, in mice (64) and non-human primates (NHP) (65), from convalescent to naïve animals, have demonstrated an important, but unsurprisingly non-sterilising, contribution of T cells in protection from SARS-CoV-2 infection. In addition, depletion of CD8^+^ T cells in NHPs with low antibody titres facilitates breakthrough infections (65), which indicates that T cell memory will have an important role in limiting disease severity and viral loads of reinfection (66) or vaccine breakthrough with antibody waning. Indeed, post-infection antibody responses wane considerably with time, resulting in retention of only 36% of initial S antibody levels and 7% N antibody levels at 1 year post-infection (67), and 10% of infected individuals do not seroconvert however manage to make long-term stable T cell responses (68). In a large-scale patient cohort study of health care workers (*n* = 285 infected (RT-PCR+) of 2,826 subjects over 200 days), higher magnitude T cell response coupled with moderate antibody responses associates with reduced risk of reinfection (66). The magnitude of ORF1a/b-, but not S or N, specific SARS-CoV-2 T cell responses during infection of adults does not differ with symptom severity but does associate with reduced duration of illness (20) indicating a pre-existing crossreactive T cell response that may play a role in mitigating COVID-19.

### SARS-CoV-2 T_FH_ Recruitment for Early Antibodies

A delayed antibody response is associated with COVID-19 severity and can be fatal (69), which can be indicated by high viral loads and uncontrolled viral replication (70). Severe COVID-19 is associated with reduced CD4^+^ and CD8^+^ T cell immunity, but not B cell responses (33). The early recruitment of CD4^+^ T cells of the T_FH_ phenotype (71) is a key cornerstone in the COVID-19 battle and correlates with antibody levels at convalescence. Our early COVID-19 case study (1) revealed that both ICOS^+^PD-1^+^CD4^+^ T_FHs_ and activated CD38^+^HLA-DR^+^ CD4^+^/CD8^+^ T cells appeared transiently in patient's blood at 3 days prior to recovery, suggesting involvement of T cells in the resolution of COVID-19. These findings were subsequently confirmed in large acute and convalescent COVID-19 cohorts (71, 72). T_FH_ cells are of a particular importance as they play an essential role in generation of antibodies, their affinity maturation, and B cell memory durability (73). Furthermore, circulating CXCR5^+^ T_FH_ cells that emerge transiently in human blood following a broad range of viral infection and vaccination, including influenza (74–76), yellow fever (77), HIV-1 (78), and Ebola (79), have a greater capacity for IL-21 and IL-10 production, leading to superior B cell helper responses (80). Delayed early recruitment of T_FH_ recruitment is therefore a prognostic indicator of COVID-19.

### T Cell Responses and Vaccine Platforms

A range of COVID-19 vaccines have been developed and clinical trials have progressed to deliver over 5 billion doses, all within 18 months of discovery of this novel virus. The most widely used vaccine platforms encode the Spike protein only either by mRNA lipoparticles [(Moderna (mRNA-1273) and Pfizer (BNT162b2)], viral vectored vaccines [Adenovirus (Ad) 26, Ad5 and Chimpanzee Adenovirus Oxford 1 (ChAdOx1)], or recombinant protein with adjuvant (NVX-CoV2373, Spike recombinant protein with M adjuvant by NovaVax), or whole inactivated SARS-CoV-2 virions (CoronaVac, Alum adjuvanted β-propiolactone inactivated vaccine). Phase I immunogenicity trials have some limited reporting on the T cell response elicited by vaccination for mRNA vaccines (36), ChAdOx1 nCoV-19 (37), NVX-CoV2373 (38), Ad26.COV2.S (39), and CoronaVac (40). Parallel comparison of different vaccine platforms in non-human primates [reviewed in (81)] shows that vaccination induces strong CD4^+^ and somewhat lower CD8^+^ T cell responses (Table 1). There are limited studies that have directly compared cellular responses of different vaccines by simultaneous parallel assessment of mRNA vaccines [Moderna (mRNA-1273) and Pfizer (BNT162b2) (36)] and inactivated vaccines (CoronaVac and BNT162b2) (82). By design, mRNA and viral vector vaccines have access to MHC class I processing machinery which may more effectively prime T cell responses; however, inactivated vaccines are also able to elicit SARS-CoV-2-specific T cells (40). Parallel comparison of CoronaVac and BNT162b2 showed inactivated vaccines recruited T cells to a similar and even greater extent as mRNA vaccines in a small study in Hong Kong, where the magnitude of S-specific CD4^+^ and CD8^+^ T cells and number of responders were comparable between mRNA and inactivated vaccine platforms at memory timepoints (82). Therefore, different vaccine platforms each elicit T cell immunity but prioritization between platforms is relatively unknown.

### Augmenting T Cell Responses During COVID-19 Vaccination

Prior infection and subsequent vaccination can result in “hybrid immunity,” whereby T cell responses are higher than either vaccination or infection alone (41) and can also result in wider antigenic breadth for antibodies (83) (Table 1). T cell responses established by prior infection are increased early after one dose of mRNA vaccination, but by the memory phase and after the second booster, CD4^+^ T cell responses are comparable between groups, whilst CD8^+^ T cell responses are increased at multiple timepoints with prior exposure long term after vaccination (42). The lag in recruitment of CD8^+^ T cells during acute SARS-CoV-2 infection (22) and 2-dose vaccination (42), but bolstered response with booster vaccination with prior infection highlights the difficulty in recruiting naïve antigen-specific CD8^+^ T cells, and vaccines may need to be more immunogenic to establish SARS-CoV-2-specific CD8^+^ T cell responses.

Heterologous boosting augments immune responses, whereby the prime-boost regimes with different vaccine platforms can have a synergistic effect compared with homologous vaccination alone, as our immune system benefits from variety that may prime and activate a wider breath of immune cells. Due to changes in public health policy and vaccine availability, some mix-and-match COVID-19 vaccine schedules have occurred [reviewed in (84)]. However, the order of heterologous vaccination is important for hybrid immunity to occur. In Sweden, health care workers who had initially been vaccinated with ChAdOx1 nCoV-19 and then received either ChAdOx1 nCoV-19 or BNT162b2 had higher magnitude neutralizing antibodies and T cells, whilst BNT162b2 then ChAdOx1 nCoV-19 vaccination did not augment responses (85, 86). Therefore, a wider prime of cellular immunity by viral vector vaccine first and then a focused mRNA vaccination may be a more synergistic vaccine regime. Furthermore, cellular immunity from vaccination can also be increased by an increased interval between vaccine doses depending on first-dose efficacy (87) and increased dose concentration (mRNA-1273 Moderna) (88). In addition, long-term vaccine efficacy is maintained to a greater extent by viral vector vaccines of DNA viruses (ChadOx1 and Ad26) compared with the short half-life of mRNA vaccines (89), which may be attributable to antigen persistence to drive long-term retention of T cell resident memory and B cell memory. Further experiments are needed to define these real-world observations for differences in cellular responses and to define future vaccine regimes as booster vaccination becomes inevitable for controlling SARS-CoV-2.

IFN-γ-producing T cell responses toward SARS-CoV-2 immunization were first defined for the phase I clinical trials. In addition to neutralizing antibodies, the numbers of SARS-CoV-2-specific IFN-γ^+^ T cells were found to increase as a consequence of the intramascular vaccination with the Moderna VRC mRNA-1273 at d43 (90), CanSino AdV5 COVID-19 at d28 (91), and Oxford/AstraZeneca ChAdOx1 nCoV19 at d14, d28, and d56 after priming (92), as detected by flow cytometry and the IFN-γ ELISpot assay. Comparing with the baseline, an approximate 10-fold increase of IFN-γ-producing T cells was observed after CanSino AdV5 vaccination (91), comparable to the levels of IFN-γ-producing T cell found after SARS-CoV-2 infection. In agreement with findings from patients with COVID-19, IFN-γ^+^CD4^+^ T cells dominated over IFN-γ^+^CD8^+^ T cells after SARS-CoV-2 Moderna mRNA-1273 immunization (90). It is most likely thought that SARS-CoV-2-specific CD4^+^ and CD8^+^ T cells primed by the vaccines can be recruited more rapidly and in much bigger numbers after subsequent SARS-CoV-2 exposure when compared to primary T cell responses. In-depth understanding of T cell-mediated immunity elicited after SARS-CoV-2 vaccination together with recruitment of those T cells after subsequent infection is needed if we are to optimize vaccine strategies and fully understand the impact of T cells on disease outcome.

Subsequent studies using peptide-HLA multimers to define SARS-CoV-2-specific T cell responses provide now enough evidence to show that robust CD4^+^ and CD8^+^ T cells can be elicited during BNT162b2 mRNA COVID-19 vaccination. The BNT162b2 vaccine elicits robust anti-S CD4^+^ T cell responses directed at the HLA-DPB1^*^04/S_166−180_ epitope in both peripheral blood and lymph nodes (93). In the peripheral blood, both CD4^+^S_166−180_ tetramer^+^ T cells of primarily CCR7^−^CD45RA^−^ effector memory phenotype and S_166−180_ tetramer^+^CXCR5^+^PD1^+^ circulating T_FH_ cells were readily detected. CD4^+^S_166−180_ tetramer^+^ T cells were found at d21 following the first-dose vaccine, peaked at d28 (7 days after the second dose) and persisted for up to 200 days. Using fine needle aspiration of draining axillary lymph nodes in 14 individuals before and after vaccination, T_FH_ cell responses were detected in lymph nodes at 30 days after the second vaccine dose and persisted for more than 170 days. The above data provide clear evidence for establishment of long-term immunological epitope-specific CD4^+^ T cell memory following BNT162b2 mRNA COVID-19 vaccination.

Promising findings revealed that the BNT162b2 mRNA COVID-19 vaccination can elicit prominent tetramer-specific CD8^+^ T cell responses toward several epitopes, including HLA-A^*^01:01, HLA-A^*^02:01, HLA-A^*^24:02, HLA-B^*^15:01, HLA-B^*^35:01, and HLA-B^*^40:02 (28, 94). An elegant study from Minervina et al. (28) used 18 DNA-barcoded MHC class I multimers for HLA-A^*^01:01, HLA-A2^*^01, HLA-A^*^24:02, HLA-B^*^15:01, and HLA-B^*^40:02, together with scRNAseq and scTCRseq to further characterize and compare tetramer-specific CD8^+^ T cell responses in patients with COVID-19 and vaccinees. The authors found that S-specific CD8^+^ T cells displayed comparable magnitude, phenotype, and also TCRαβ repertoire diversity and TCR motifs after both vaccination and infection, indicating the robustness of tetramer-binding CD8^+^ T cell responses elicited after COVID-19 mRNA vaccines. Importantly, prominent SARS-CoV-2 CD8^+^ T cells were found in both naïve and recovered individuals after mRNA vaccination, with the latter showing more of a CCR7^−^CD45RA^+^ effector phenotype.

### Epitope Conservation and T Cell Crossreactivity for VoC

The nsp14 of SARS-CoV-2 operates as a proofreading endonuclease, which is a common feature of CoVs; therefore, the evolutionary rate of adaptation is lower for SARS-CoV-2 than other viruses such as seasonal influenza viruses which are notorious for antigenic drift. T cell memory is remarkably stable long term, and 17 years after SARS-CoV infection, antigen-specific T cells can be detected (20). Therefore, given the relative genomic stability of SARS-CoV-2 and therefore epitope conservation and T cell longevity, it is anticipated that T cell responses will provide a long-term protective barrier against severe disease once established.

However, due to the widespread number of infections of the pandemic, numerous SARS-CoV-2 lineages have emerged. VoCs with distinct serological profiles and varying antigenic distance from the ancestral Wuh1 strains may undermine infection and vaccination derived immunity to some extent. Comparisons of reduction in transmission rates post-vaccination against Alpha VoC (B.1.1.7) and Delta VoC (B.1.617.2) have found that there is a decline in reduced transmission with B.1.617.2 infection, compared with B.1.17, after two doses of BNT162b2 or ChAdOx1 vaccination (95). This decline in protection from contact tracing of infection with time post-vaccination was less pronounced in BNT162b2 vaccinated individuals' short term after vaccination. However, despite reduced neutralization of some VoC, the T cell response has maintained high levels of crossreactivity. In Pfizer/BioNTech BNT162b2 mRNA-vaccinated health care workers, Wuh1-S-specific CD4^+^ T cells are crossreactive for VoCs Alpha (B.1.351) and Beta (B.1.1.7); however, antibody responses are only partially crossreactive in terms of neutralization (47) and FcR functions (96). Furthermore, in COVID-19 convalescents and vaccine recipients of the Moderna (mRNA-1273) or Pfizer/BioNtech (BNT162b2) COVID-19 vaccines, the original CD4^+^ and CD8^+^ T cell responses were comparable against the B.1.1.7, B.1.351, P.1, and CAL.20C lineages; however, amino acid epitope conservation was reduced in 7% of CD4^+^ T cell epitopes and 3% of CD8^+^ T cell epitopes (36, 39) (Table 1). Therefore, whilst T cell reactivity is relatively maintained against SARS-CoV-2 VoC, with time as the antigenic space is explored, within chronically infected individuals (97) or across the population, T cell escape may occur and broader vaccine targets or strategies to bolster T cell responses may be needed.

## Final Thoughts: SARS-CoV-2 T Cells

T cell responses are a cornerstone to protective antiviral immunity, with evidence that higher baseline crossreactive responses can reduce the duration of SARS-CoV-2 infection, and patients with severe COVID-19 have reduced T cell responses. Animal T cell passive transfer studies show a non-sterilising but protective effect from T cell memory when antibody responses are limited. However, there is inefficient recruitment of T cell responses during SARS-CoV-2 infection, as evident by incomplete T cell receptor diversity compared with available naïve precursors. This may be attributable to viral factors such as ORF8-mediated MHC-I downregulation or the antiinflammatory cytokine milieu of COVID-19 leading to reduced T cell immunity. Long-term maintenance of T cell responses for 17 years following SARS-CoV infection lends hope to effectively arming T cells in the post-COVID-19 era, where bolstered hybrid immunity is evident from recovered vaccinated individuals or combination platforms of vaccines. Despite waning of neutralizing antibodies and variants of concern eroding serological responses, protection from severe disease is maintained by most vaccine platforms, which may be attributable to bolstered T cell immunity even by inactivated vaccines. The capacity of SARS-CoV-2-specific T cells to maintain recognition of variants of concern due to epitope conservation by the viral proofreading endonuclease may enable long-term protection from severe COVID-19 disease.

## Author Contributions

SV conceptualized the review. All authors contributed to the first draft, final review, article, and approved the submitted version.

## Funding

This study was partly supported by the Theme-based Research Grants Scheme (T11-712/19-N) and Health and Medical Research Fund (HMRF COVID-190115 and COVID-190126). This work was supported by the NHMRC Leadership Investigator Grant to KK (1173871), NHMRC Emerging Leadership Level 1 Investigator Grant to THON (#1194036).

## Conflict of Interest

The authors declare that the research was conducted in the absence of any commercial or financial relationships that could be construed as a potential conflict of interest.

## Publisher's Note

All claims expressed in this article are solely those of the authors and do not necessarily represent those of their affiliated organizations, or those of the publisher, the editors and the reviewers. Any product that may be evaluated in this article, or claim that may be made by its manufacturer, is not guaranteed or endorsed by the publisher.
